# A neuraminidase-targeted nanobody confers broad protection against influenza B virus

**DOI:** 10.1128/jvi.00762-26

**Published:** 2026-07-10

**Authors:** Hang Jia, Chaohui Lin, Yinghao Guo, Weigang Cai, Renfang Guan, Lizhu Zhu, Qian Yang, Huihui Kong, Bin Sun, Zeli Zhang, Jian Ma

**Affiliations:** 1 Department of Pancreatobiliary Surgery and Department of Immunology, The Second Affiliated Hospital of Harbin Medical University, Harbin Medical University 34707, Harbin, China; 2 Zhejiang Key Laboratory of Multi-Omics in Infection and Immunity, Center for Infectious Disease Research, School of Medicine, Westlake University 681395, Hangzhou, China; 3 Worldwide Influenza Centre, The Francis Crick Institute 376570, London, United Kingdom; 4 State Key Laboratory of Animal Disease Control and Prevention, Harbin Veterinary Research Institute, Chinese Academy of Agricultural Sciences 111613, Harbin, China; 5 Research Center for Pharmacoinformatics, College of Pharmacy, Harbin Medical University 34707, Harbin, China; Emory University School of Medicine, Atlanta, Georgia, USA

**Keywords:** influenza B virus, neuraminidase, nanobody, enzymatic inhibition, antiviral therapy

## Abstract

Influenza B virus (IBV) exhibits antigenic diversity and significantly contributes to the annual burden of influenza-related disease, posing substantial impacts on global public health and the economy. In this study, we focused on the neuraminidase (NA) of IBV, immunized an alpaca with tetrameric IBV NA, and identified a series of nanobodies with a common feature of the “DYR” motif in CDR3, exhibiting broad inhibitory activity against diverse NAs of IBV. Among these, the most potent VHH5 broadly inhibits NA from multiple representative strains across ancestral, B/Yamagata/16/88-like, and B/Victoria/2/87-like lineages. Moreover, fusing VHH5 to a human immunoglobulin G1 Fc domain significantly increased its antiviral potency *in vitro*, with the 50% inhibitory concentration in the low nanomolar range. Structural modeling and molecular dynamics simulations revealed that the “DYR” motif within the elongated CDR3 loop of VHH5 penetrates the catalytic pocket and engages the highly conserved catalytic site of IBV NA. Experimental mutagenesis analysis of the predicted interface further supported this binding model. Furthermore, the VHH5-Fc protects mice from the lethal challenge with a high dose of the IBV-2024 virus in both prophylactic and therapeutic settings. Our findings highlight the potential of the Fc-fused VHH5 antibody in controlling influenza B virus infections.

**IMPORTANCE:**

Although influenza B viruses (IBVs) account for a substantial proportion of seasonal influenza virus infections, they have received comparatively less research attention. The small size and flexible binding modes of nanobodies enable them to access cryptic or recessed epitopes, supporting their development as potent clinical therapeutics. Here, we report the identification of VHH5, a nanobody targeting conserved residues within the neuraminidase active site of the influenza B virus, conferring broad inhibitory activity. Moreover, Fc-fused VHH5 enhanced antiviral potency *in vitro* and provided robust protection against lethal IBV challenge in mice. Together, these findings establish VHH5-Fc as a promising therapeutic candidate with potent and broad-spectrum activity against antigenically diverse IBV strains.

## INTRODUCTION

Seasonal influenza virus is a prevalent respiratory pathogen that can cause severe respiratory illness, resulting in approximately 1 billion infections and 300,000 to 600,000 respiratory-related deaths annually, with the influenza B virus (IBV) accounting for approximately 20 to 30% of these fatalities (1, 2). First identified in 1940, IBVs diverged into two antigenically and genetically distinct lineages, the B/Yamagata/16/88-like and the B/Victoria/2/87-like lineages (3–5). Since 1983, these lineages have co-circulated globally, collectively contributing to around 25% of human influenza cases between 2000 and 2018 (6). Notably, the B/Yamagata/16/88-like lineage viruses have not been detected worldwide since 2020, indicating a significant shift in the epidemiology of IBV (7, 8).

Although IBV infections generally produce milder symptoms compared to those caused by influenza A virus (IAV), they are associated with a higher risk of severe outcomes among hospitalized patients, particularly in children and the elderly (9–11). A cohort study conducted from 2004 to 2013 revealed that hospitalized patients aged ≤16 years with IBV infection had higher mortality rates than those infected with IAV (12). Moreover, healthy children aged ≥10 years with IBV faced a significantly greater risk of intensive care unit admission compared to their counterparts with IAV (12). During the IBV-dominant 2017–2018 winter season, the EuroMOMO network reported elevated excess mortality among the elderly across Europe (11).

Hemagglutinin (HA) and neuraminidase (NA) are the two major glycoproteins of influenza viruses, playing critical roles in the viral infection cycle. Currently licensed seasonal influenza vaccines primarily target the HA protein but exhibit suboptimal effectiveness, ranging from 10% to 60% (13). In contrast to HA, NA evolves more slowly and harbors a highly conserved enzymatic active site that is essential for efficient viral release from infected cells, making it a promising target for the development of vaccines and antiviral therapeutics (14–16). Since 2010, several NA inhibitors, including oseltamivir, peramivir, and zanamivir, have been widely used to treat influenza virus infections (17). However, the clinical efficacy of these inhibitors against IBV is generally lower than against IAV (18). Furthermore, the emergence of various NA mutations in IBV has led to significant, and in some cases severe, resistance to existing neuraminidase inhibitors (NAIs), compromising efforts in IBV prevention and treatment (17). Previous studies have reported broadly protective murine monoclonal antibodies against influenza B virus NA (19). Likewise, human monoclonal antibodies targeting IAV NA have demonstrated robust protective efficacy in animal models, further supporting NA as a viable target for antiviral antibody development (20–22).

Camelid variable heavy chain domain antibodies, known as nanobodies, are single-domain antibodies characterized by a small size and an extended, flexible complementarity-determining region 3 (CDR3) (23–25). This distinctive structural feature enables nanobodies to form prominent loop structures that can access cryptic or conformationally constrained epitopes, which are often inaccessible to conventional immunoglobulin G (IgG) antibodies (25, 26). Given the deep and narrow catalytic pocket of IBV NA, we hypothesized that the extended CDR3 of nanobodies may confer superior accessibility to the enzyme’s active site. In this study, we report the isolation of a series of nanobodies characterized by a conserved “DYR” motif within their extended CDR3, exhibiting broad inhibitory activity against diverse NAs of IBV. Among these, VHH5 demonstrates the highest potency, and the VHH5-Fc fusion protein exhibits broad inhibitory activity *in vitro*, providing protection against a lethal challenge with high-dose IBV-2024 *in vivo*.

## RESULTS

### Identification of potent nanobodies targeting IBV NA from an immune VHH library

**Fig 1:**
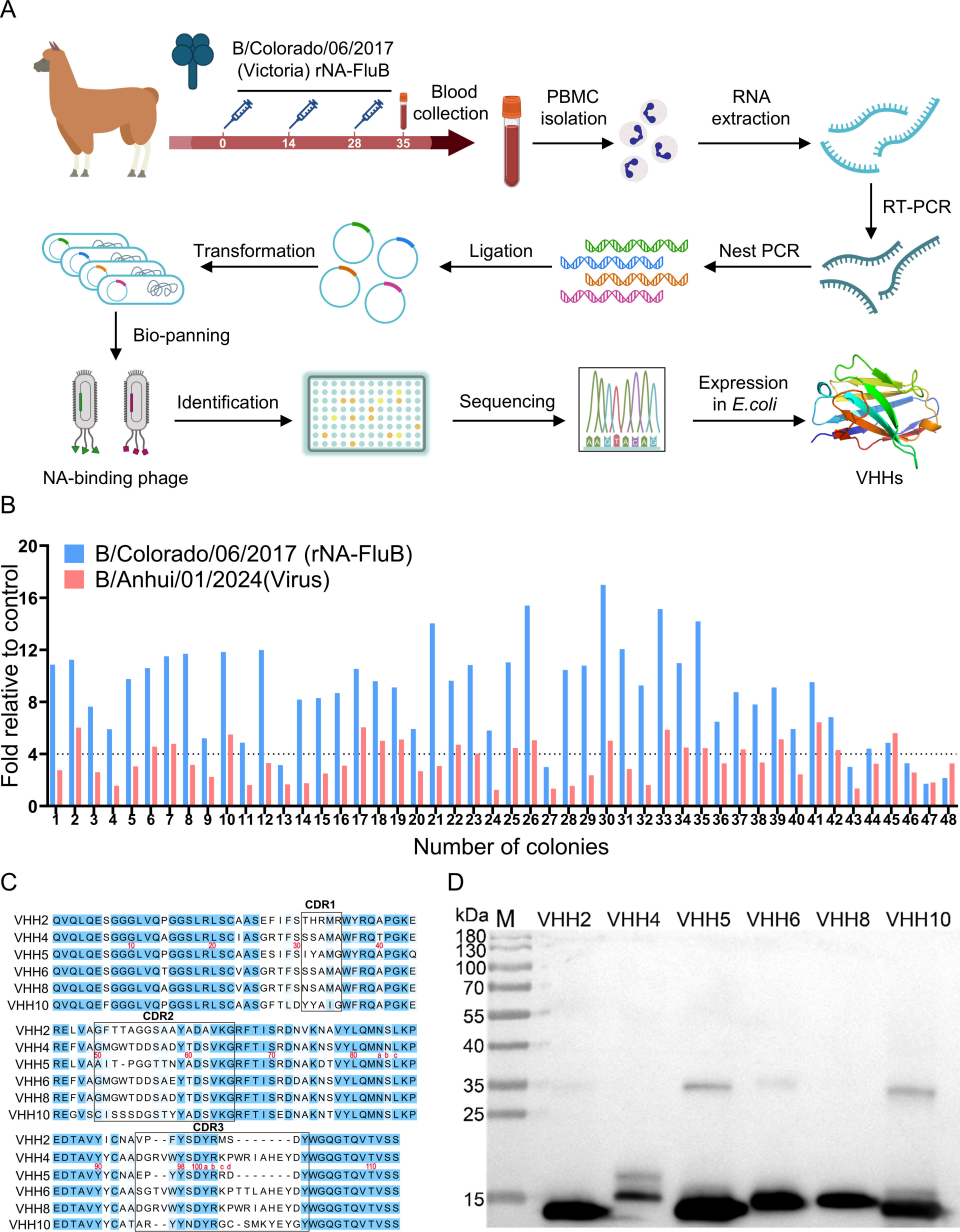
Identification of influenza B neuraminidase-specific nanobodies from an immunized alpaca. (**A**) Schematic overview of the VHH discovery workflow. An alpaca was immunized with recombinant neuraminidase (rNA-FluB) from B/Colorado/06/2017 (B/Victoria/2/87-like lineage). After PBMC isolation, RNA extraction, and reverse transcription, the VHH-encoding sequences were PCR-amplified and ligated into the pComb3X phagemid vector for phage display library construction. After bio-panning, NA-binding phages were identified, sequenced, and expressed in *Escherichia coli* as soluble VHHs. (**B**) Individual colonies were randomly selected and screened for reactivity to rNA-FluB and virus-derived NA (B/Anhui/01/2024, the B/Victoria/2/87-like lineage) via phage ELISA. The dashed line represents the positivity threshold. Clones exhibiting OD₄₅₀ values at least fourfold higher than those of PBS-coated controls for both antigens were considered positive. (**C**) Amino acid sequence alignment of the six NA-specific nanobodies, with CDRs annotated according to the Kabat numbering scheme and highlighted in boxed regions. (**D**) Western blot analysis of recombinant VHHs expressed in *E. coli*.

To isolate IBV NA-specific VHHs, a healthy female alpaca was immunized three times at 2 week intervals with recombinant NA protein (rNA-FluB) derived from B/Colorado/06/2017 (B/Victoria/2/87-like lineage), formulated with Freund’s adjuvant (Fig. 1A and Fig. S1A). Seroconversion was confirmed by day 35, as immune serum exhibited strong binding to rNA-FluB at a dilution of 1:32,000, indicative of a robust humoral immune response against NA (Fig. S1B). Peripheral blood mononuclear cells (PBMCs) were collected on day 35, followed by total RNA extraction and cDNA synthesis via reverse transcription (Fig. 1A). A phage display library was constructed and subjected to three rounds of solid-phase bio-panning against rNA-FluB. The phage output-to-input ratio increased from 1.1 × 10⁻^7^ in the first round to 4 × 10⁻^5^ in the third round, demonstrating significant enrichment of NA-reactive clones (Fig. S1C).

From the third round, 48 clones were randomly selected and screened for reactivity against both rNA-FluB and virus-derived NA (B/Anhui/01/2024). As shown in Fig. 1B, 20 clones exhibited specific binding, defined as an OD_450_ value exceeding four times that of phosphate-buffered saline (PBS)-coated control wells for both NA antigens. These specifically binding clones were subsequently selected for sequencing. As shown in Fig. 1C, a total of six unique sequences were identified, namely VHH2, VHH4, VHH5, VHH6, VHH8, and VHH10. The expression and purity of the corresponding nanobodies were assessed by Western blotting and sodium dodecyl sulfate–polyacrylamide gel electrophoresis (SDS-PAGE), which showed a predominant band at the expected molecular weight (~15 kDa), confirming high purity after purification (Fig. 1D and Fig. S1D). Collectively, these results demonstrate the successful generation of a high-quality immune VHH library and the identification of a series of VHHs targeting the influenza B virus NA.

### Binding activity and cross-lineage inhibitory effects of nanobodies against influenza B virus NA

**Fig 2:**
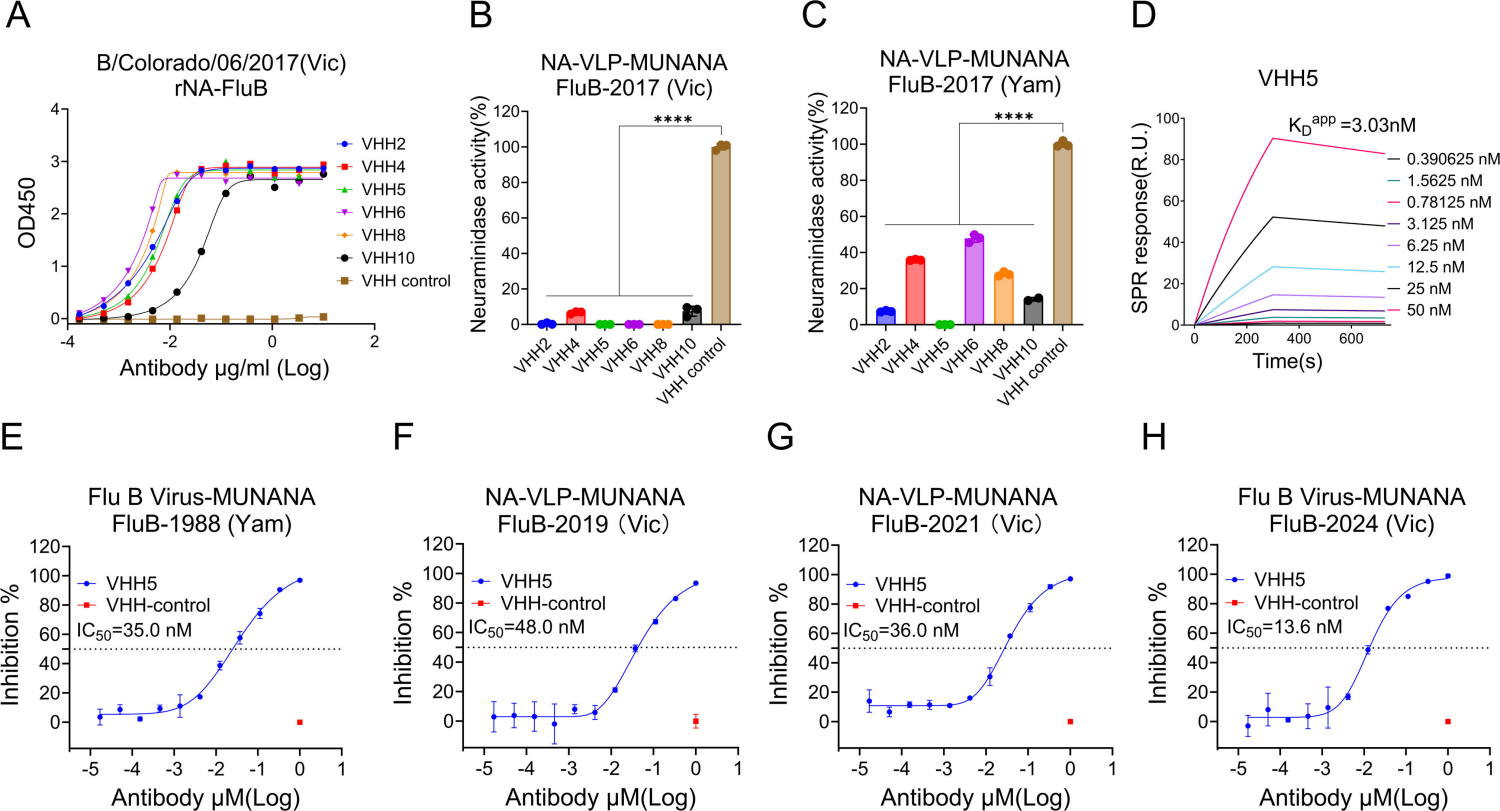
Functional characterization of influenza B virus NA-specific nanobodies. (**A**) Binding curves of nanobodies to rNA-FluB were determined by ELISA. (**B and C**) The inhibitory activity of VHHs at 10 µg/mL against NA-VLPs derived from representative influenza B virus strains were measured using the MUNANA assay. FluB-2017 (Vic) and FluB-2017 (Yam) denote B/Colorado/06/2017 (B/Victoria/2/87-like lineage) and B/New York City/PV00094/2017 (B/Yamagata/16/88-like lineage), respectively. (**D**) The apparent binding affinity of VHH5 to rNA-FluB was measured by SPR. (**E–H**) NA inhibition curves of VHH5 against representative IBV NAs were measured using the MUNANA assay. FluB-1988 (Yam), FluB-2019 (Vic), FluB-2021 (Vic), and FluB-2024 (Vic) denote B/Yamagata/16/1988 (B/Yamagata/16/88-like lineage), B/Washington/02/2019 (B/Victoria/2/87-like lineage), B/Michigan/01/2021 (B/Victoria/2/87-like lineage), and B/Anhui/01/2024 (B/Victoria/2/87-like lineage), respectively. The VHH control was included at the highest tested concentration as a negative control. The dashed line indicates the IC_50_. All data are normalized to a control nanobody (VHH control) and presented as mean ± SD. Statistical significance was determined by two-tailed unpaired Student’s *t*-tests. ****P < 0.0001.

To evaluate the binding ability of six VHHs to IBV NA, we conducted an enzyme-linked immunosorbent assay (ELISA). Compared to the negative control VHH, all six VHHs displayed strong reactivity to rNA-FluB (Fig. 2A). To determine whether these nanobodies target the conserved catalytic active site of IBV NA, we performed a 2′-(4-methylumbelliferyl)-α-D-N-acetylneuraminic acid (MUNANA) assay using NA virus-like particles (VLPs) produced by co-expressing human immunodeficiency virus type 1 gag, pol, and Rev with NA derived from IBV in human embryonic kidney (HEK) 293T cells. The results indicated that all six VHHs exhibited varying degrees of inhibitory activity against NA-VLPs from both the B/Victoria/2/87-like (2017) and B/Yamagata/16/88-like (2017) lineages (Fig. 2B and C). Notably, VHH5 demonstrated the most potent inhibition and was therefore selected for further functional characterization. Using the surface plasmon resonance (SPR) assay described in the Materials and Methods, we measured “apparent” binding affinities (K_D_^app^) of 3.03 nM, indicative of a high-affinity interaction between VHH5 and rNA-FluB under the experimental conditions (Fig. 2D and Fig. S1E). We next evaluated the inhibitory breadth of VHH5 against representative IBV NAs using the MUNANA assay in a dose-response format. VHH5 inhibited NA activity from both the B/Yamagata/16/88-like lineage (1988) and the B/Victoria/2/87-like lineage (2019, 2021, and 2024) in a concentration-dependent manner, with 50% inhibitory concentration (IC₅₀) values ranging from 13.6 to 48.0 nM. In contrast, the VHH control showed no detectable inhibition. These results demonstrate that VHH5 retains broad nanomolar NA-inhibitory activity against the tested IBV NAs (Fig. 2E through H). Collectively, these results demonstrate that VHH5 is a broadly inhibitory nanobody targeting IBV NA, underscoring its potential as a candidate antiviral agent.

### Engineering VHH5 into Fc-fusion formats enhances its NA inhibitory activity and antiviral efficacy

**Fig 3:**
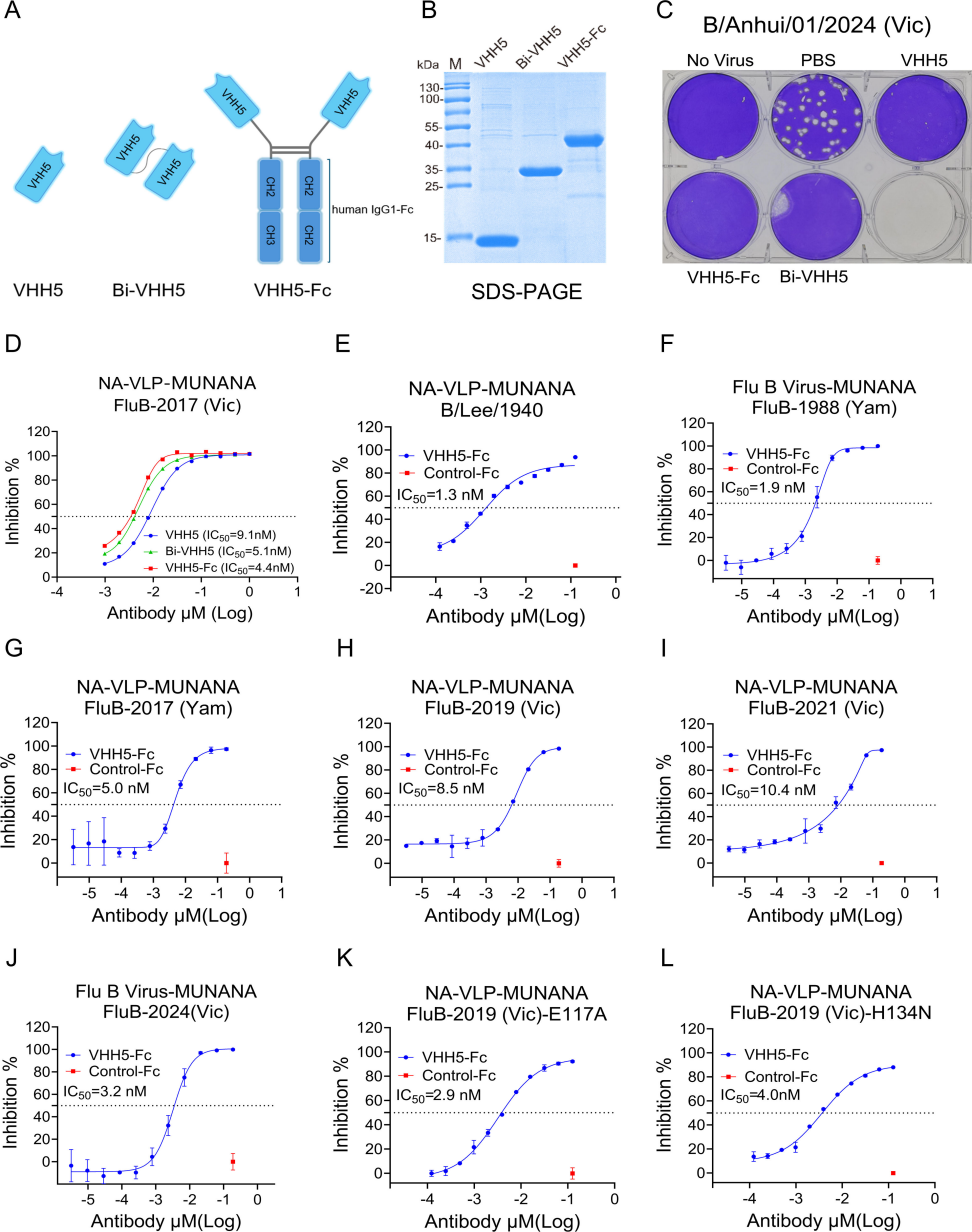
Generation and validation of bivalent and Fc-fused VHH5. (**A**) Schematic illustration of monovalent VHH5, Bi-VHH5, and human IgG1 Fc-fused VHH5 (VHH5-Fc). (**B**) SDS-PAGE analysis of purified VHH5, Bi-VHH5, and VHH5-Fc visualized by Coomassie Brilliant Blue staining. (**C**) The inhibition capacity of VHH5, Bi-VHH5, and VHH5-Fc against FluB-2024 (Vic) was measured by a plaque reduction assay, with each nanobody applied at a concentration of 1 µM in the overlay medium. (**D**) NA inhibition curves of VHH5, Bi-VHH5, and VHH5-Fc against NA-VLPs derived from FluB-2017 (Vic) were measured using the MUNANA assay. (**E–J**) NA inhibition curves of VHH5-Fc against B/Lee/1940 (ancestral), FluB-1988 (Yam), FluB-2017 (Yam), FluB-2019 (Vic), FluB-2021 (Vic), and FluB-2024 (Vic) were measured using the MUNANA assay. (**K and L**) NA-inhibitory activity of VHH5-Fc against B/Washington/02/2019 NA-VLPs carrying the NAI resistance-associated E117A (**K**) or H134N (**L**) substitution was measured using the MUNANA assay. The control-Fc was included at the highest tested concentration as a negative control. The dashed line indicates the IC₅₀.

To enhance antiviral potency, nanobodies are commonly engineered into bivalent or Fc-fused formats to compensate for their small size. In this study, a bivalent VHH5 (Bi-VHH5) was generated by linking two VHH5 domains via a flexible (GGGGS)₃ peptide linker, while VHH5-Fc was constructed by fusing the C-terminus of VHH5 to human IgG1 Fc domain (Fig. 3A). SDS-PAGE analysis confirmed expression of proteins with the expected molecular weights: approximately 15 kDa for monomeric VHH5, 30 kDa for Bi-VHH5, and 40 kDa for VHH5-Fc (Fig. 3B). To assess their ability to inhibit viral replication, plaque assays were performed with antibodies incorporated into the overlay medium, as anti-NA antibodies block viral release during budding rather than viral entry. At a concentration of 1 μM, both Bi-VHH5 and VHH5-Fc effectively suppressed plaque formation, whereas monomeric VHH5 only partially reduced plaque numbers (Fig. 3C). We next compared the NA-inhibitory activity of the three VHH5 formats using the MUNANA assay. Both Bi-VHH5 and VHH5-Fc retained the ability to inhibit IBV NA enzymatic activity. Among the three formats, Bi-VHH5 and VHH5-Fc showed comparable IC₅₀ values, approximately twofold lower than VHH5 (Fig. 3D). Given its superior performance, VHH5-Fc was selected for further evaluation in a MUNANA assay panel. VHH5-Fc inhibited NA activity in a concentration-dependent manner when tested against NAs from B/Lee/1940 (ancestral), the B/Yamagata/16/88-like lineage (1988 and 2017), and the B/Victoria/2/87-like lineage (2019, 2021, and 2024), with IC₅₀ values in the nanomolar range (Fig. 3E through J). We next evaluated the inhibitory activity of VHH5-Fc against NAI-resistant NA variants using B/Washington/02/2019 NA-VLPs carrying either the E117A or H134N substitution, which have been associated with reduced susceptibility to several small-molecule NAIs in previous studies (27, 28) (Fig. 3K and L). VHH5-Fc retained concentration-dependent inhibitory activity against both mutant NA-VLPs, indicating that these substitutions do not abolish its NA-inhibitory function. We also attempted to evaluate VHH5-Fc against NA-VLPs bearing the Wuhan spiny eel influenza virus (WSEIV) NA, a divergent influenza B-like virus (29). Under the assay conditions tested, VHH5-Fc did not show detectable inhibitory activity against WSEIV NA (Fig. S1F). Collectively, these results indicate that VHH5-Fc maintains broad NA-inhibitory activity against representative IBV NAs and the NAI-resistant NA variants tested.

### Protective efficacy of VHH5-Fc against seasonal influenza B virus infection *in vivo*

**Fig 4:**
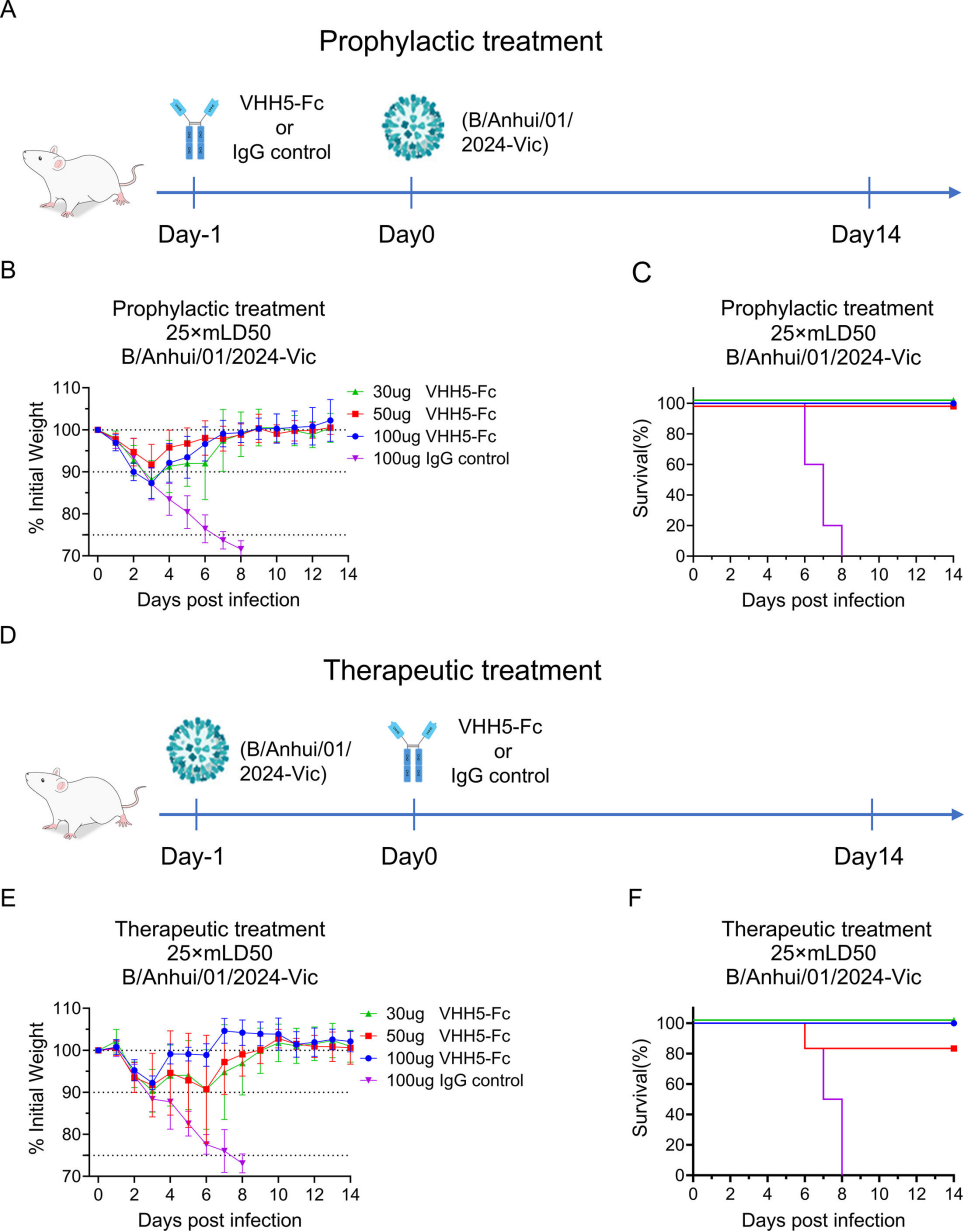
*In vivo* protection by prophylactic and therapeutic treatment with VHH5-Fc. (**A–C**) Experimental design for prophylactic administration of VHH5-Fc. Female BALB/c mice received intraperitoneal injections of VHH5-Fc or IgG control 24 h prior to intranasal challenge with 25× mLD50 of B/Anhui/01/2024. The VHH5-Fc treatment groups received doses of 30 µg (*n* = 12), 50 µg (*n* = 11), or 100 µg (*n* = 14); the IgG control group received 100 µg (*n* = 12). Graphs depict percentage body weight change (**B**) and survival curves (**C**) following prophylactic treatment. (**D–F**) Experimental design for therapeutic administration of VHH5-Fc. Female BALB/c mice were challenged intranasally with 25× mLD50 of B/Anhui/01/2024 and subsequently treated intraperitoneally with VHH5-Fc or IgG control 24 h post-infection. Treatment groups received 30 µg (*n* = 6), 50 µg (*n* = 6), or 100 µg (*n* = 7) of VHH5-Fc; the IgG control group received 100 µg (*n* = 6). Graphs show the percentage change in body weight (**E**) and survival curves (**F**) following therapeutic treatment.

*In vitro* studies demonstrate that VHH5-Fc exhibits potent NA inhibitory activity and effectively suppresses viral replication. However, *in vitro* findings alone are insufficient to fully predict antibody performance in a complex physiological environment. To evaluate the *in vivo* protective efficacy of VHH5-Fc against a high-dose challenge with IBV, we established both prophylactic and therapeutic administration models. Mice received intraperitoneal injections of varying doses of VHH5-Fc either 24 h before or 24 h after intranasal challenge with 25× 50% mouse lethal dose (mLD50) of the B/Anhui/01/2024 virus, followed by daily monitoring of body weight and survival outcomes (Fig. 4A and D). In the prophylactic setting, all VHH5-Fc-treated mice survived without mortality and experienced only transient weight loss (~10%) between days 1 and 3, with full recovery by day 4, in contrast to progressive weight loss and high mortality in the control group (Fig. 4B and C). In the therapeutic setting, mice treated with IgG control exhibited continuous weight loss from days 1 to 8, whereas VHH5-Fc-treated mice began to regain weight by day 3 (Fig. 4E). Complete protection was observed at doses of 100 μg and 30 μg, with an 83% survival rate at the 50 μg dose (Fig. 4F). These results indicate that VHH5-Fc provides robust protection against a lethal dose of influenza B virus in mice under both prophylactic and therapeutic conditions.

### Structural basis underpinning VHH5’s potent inhibition of NA

**Fig 5:**
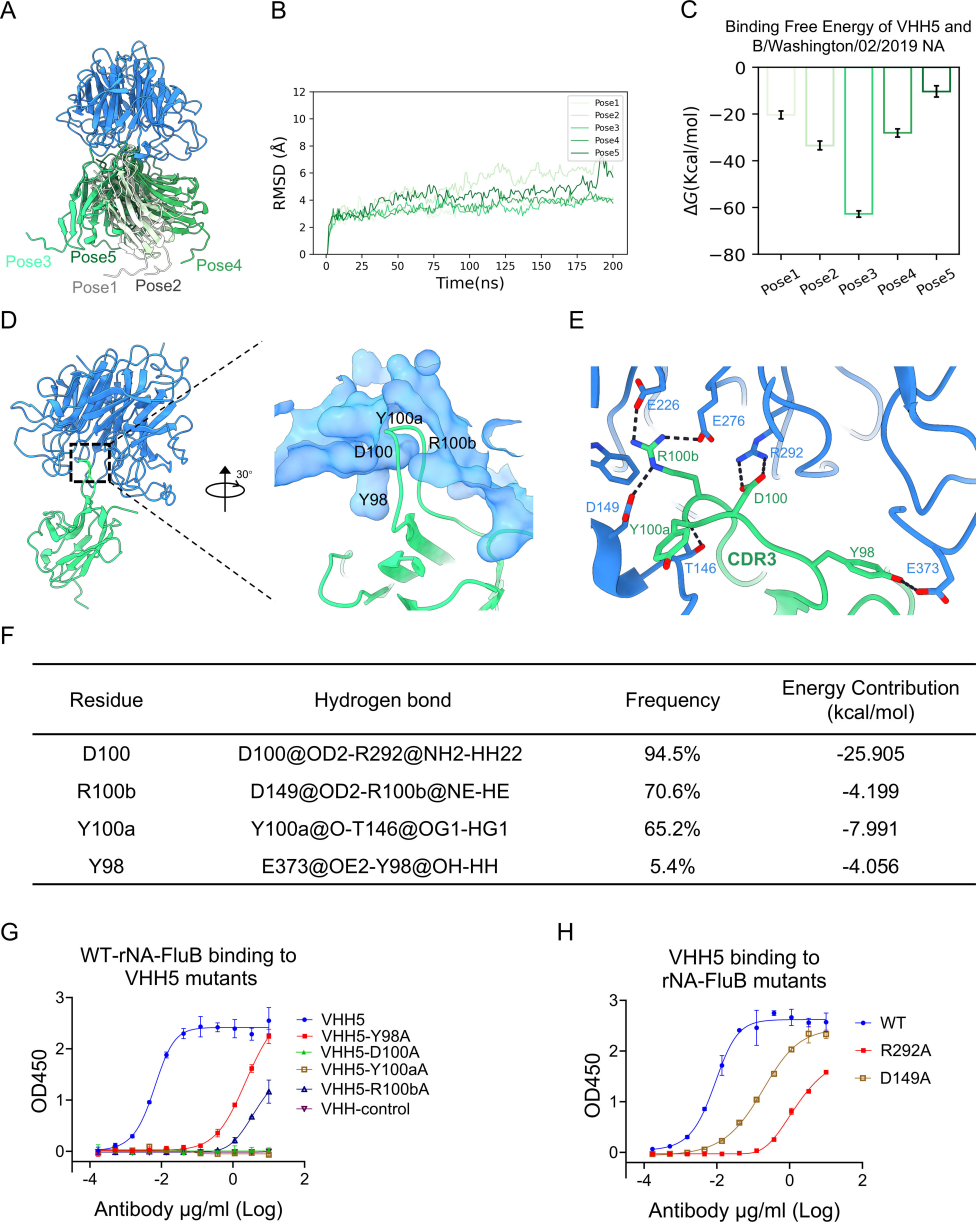
Structural basis underpinning VHH5’s potent inhibition of NA. (**A**) Five candidate docking poses of VHH5 (shown in various shades of green) bound to NA from the B/Washington/02/2019 strain (blue). (**B**) Time-dependent RMSD of the five VHH5-NA complexes from their starting structures during MD simulations. (**C**) Binding free energies for the five VHH5-NA poses, calculated by MM/GBSA. (**D and E**) Close-up view of key VHH5 residues (stick representation) inserting into the NA catalytic pocket (pose 3). Black dashed lines indicate hydrogen bonds. (**F**) Hydrogen-bond occupancy between key VHH5 and NA residues in pose 3 during the MD simulation. (**G**) Binding curves of wild-type VHH5 and alanine-substituted VHH5 mutants to rNA-FluB were determined by ELISA. (**H**) Binding curves of VHH5 to wild-type rNA-FluB and alanine-substituted rNA-FluB mutants were determined by ELISA. The R292A and D149A NA mutants were generated based on predicted interface residues with high interaction frequencies in MD simulations.

To elucidate the molecular mechanism underlying VHH5 binding to and enzymatic inhibition of IBV NA, we performed protein docking combined with molecular dynamics (MD) simulations based on the NA from B/Washington/02/2019. These analyses predicted the structural basis of the VHH5-NA interaction. The top 5 initial docking poses revealed a conserved binding pattern, with all VHH5 orientations engaging the catalytic site of NA (Fig. 5A), suggesting an intrinsic propensity of VHH5 for this region. To assess the stability of these poses and identify the most plausible binding conformation, all-atom MD simulations were conducted. Time-dependent root-mean-square deviation (RMSD) analyses demonstrated that pose 1 and pose 3 exhibited superior structural stability, as indicated by lower and more plateaued RMSD values (Fig. 5B). Subsequent molecular mechanics generalized born surface area (MM/GBSA) binding free energy calculations identified pose 3 as the most energetically favorable, with the lowest binding free energy, thereby supporting its role as the predominant binding mode (Fig. 5C). Additional MD simulations revealed that VHH5 forms stable complexes with NA proteins from both the B/Victoria/2/87-like and B/Yamagata/16/88-like lineages in the pose 3 model, with comparably low binding free energies (Fig. S2B and C). Collectively, these computational analyses support pose 3 as the predominant binding mode for VHH5.

In this configuration, the CDR3 loop of VHH5 deeply inserts into the NA catalytic pocket (Fig. 5D), establishing hydrogen bonds between VHH5 residues Y98, D100, Y100a, and R100b and NA residues E373, R292, T146, and D149, respectively (Fig. 5E). The stability and energetic significance of these interactions were further corroborated by their high formation frequencies during MD simulations and substantial per-residue contributions to the total binding free energy (Fig. 5F and Fig. S2A).

To assess the contribution of the predicted VHH5-side interfacial residues to NA binding, we compared the binding profiles of wild-type VHH5 and its alanine-substituted mutants to rNA-FluB by ELISA across a range of antibody concentrations. Compared with wild-type VHH5, the D100A, Y100aA, and R100bA mutants showed markedly reduced binding, and Y98A also exhibited reduced binding (Fig. 5G). These data support the involvement of Y98, D100, Y100a, and R100b in VHH5-mediated recognition of IBV NA.

To further evaluate the contribution of predicted NA interface residues, the binding of VHH5 to wild-type, R292A, or D149A rNA-FluB was assessed by ELISA, and concentration-dependent binding curves were generated to compare relative binding activity (Fig. 5H). Both the R292A and D149A mutants reduced VHH5 binding compared with wild-type rNA-FluB, with R292A displaying the most pronounced effect. These findings experimentally support the involvement of R292 and D149 in VHH5 recognition and are consistent with the proposed VHH5-NA binding interface.

Notably, the binding mode of VHH5 to IBV NA differs from that of the previously reported VHH525-IBV NA complex (27). Although both nanobodies primarily engage the NA catalytic pocket via their CDR3 loops, they adopt distinct overall orientations and utilize different CDR3 residues for interaction (Fig. S3A). MD simulations and buried solvent-accessible surface area analyses revealed that the VHH5-NA complex in pose 3 features a larger binding interface (Fig. S3B), with deeper penetration of the VHH5 CDR3 loop into the catalytic site. While MM/GBSA calculations indicate favorable binding free energies for both VHH5 and VHH525, repositioning VHH5 into the VHH525 binding orientation results in a marked reduction in binding affinity (Fig. S3C). These data collectively demonstrate that VHH5 preferentially adopts the pose three conformation over the VHH525-like binding mode for optimal interaction with IBV NA.

### Evolutionary diversification of IBV lineages and conserved neuraminidase residues targeted by VHH5

**Fig 6:**
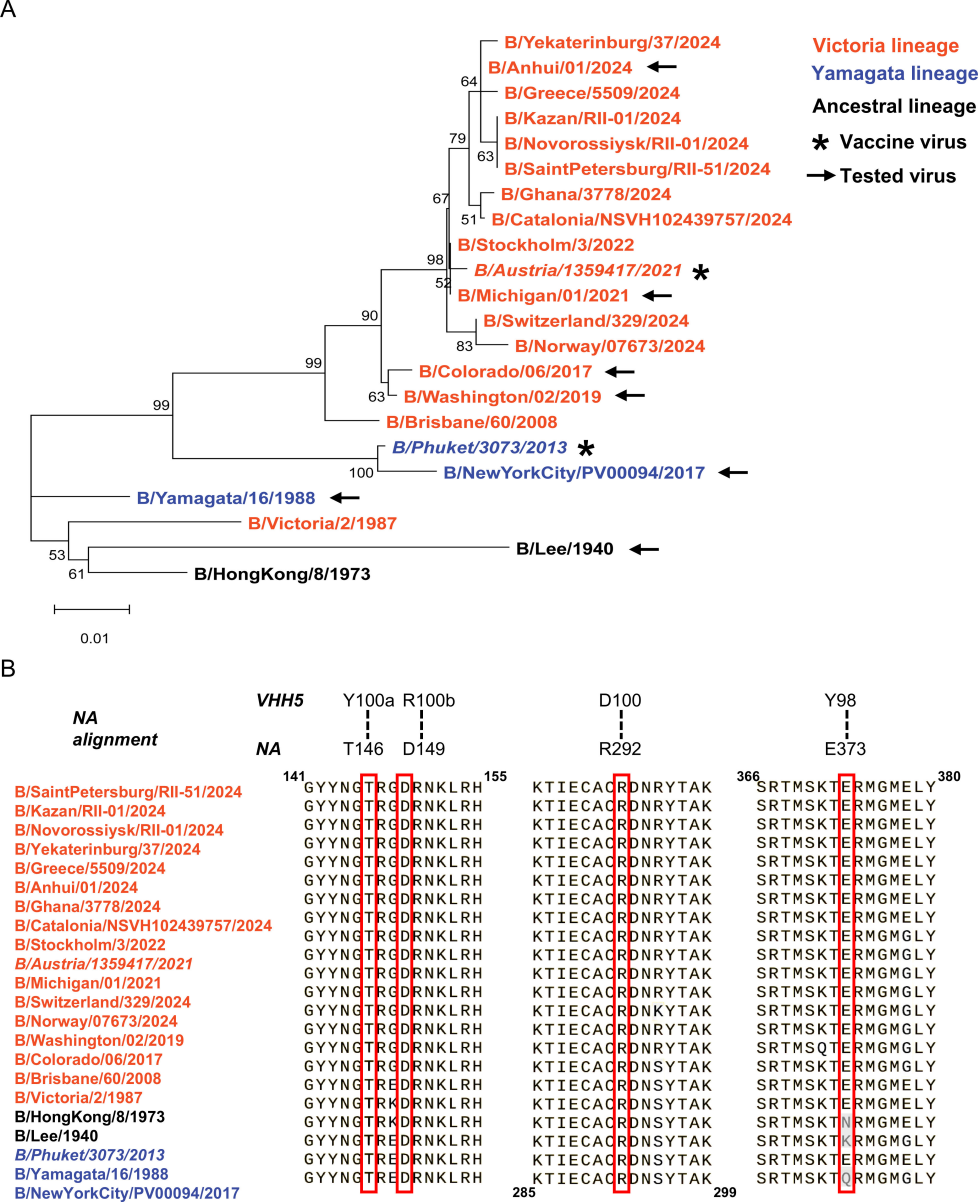
Comparison of IBVs strains. (**A**) Divergent evolutionary trajectories of NA sequences from representative influenza B viruses, including WHO-recommended vaccine strains, historical evolutionary intermediates, and recently emerged variants. The phylogeny resolves three major groups—B/Yamagata/16/88-like (blue), B/Victoria/2/87-like (red), and early diverged/ancestral strains (black). Scale bar represents amino acid substitutions per site. (**B**) Key residues within the NA catalytic site that interact with VHH5 are highly conserved among representative IBV strains. Multiple-sequence alignment of NA reveals that the four hydrogen-bonding residues involved in VHH5 binding are completely conserved across the B/Victoria/2/87-like lineage, with only a single amino acid variation at position 373 observed in some B/Yamagata/16/88-like and ancestral strains.

Phylogenetic analysis of representative IBV strains using the neighbor-joining method reproduced the expected evolutionary relationships, separating viruses into ancestral, B/Yamagata/16/88-like, and B/Victoria/2/87-like clades (30, 31), as illustrated for NA in Fig. 6A and for HA in Fig. S4A. The tested virus panel spans the ancestral strain B/Lee/1940 and representative viruses from both the B/Yamagata/16/88-like and B/Victoria/2/87-like lineages. This broad phylogenetic representation confirms that the tested virus panel encompasses the major IBV lineages, further demonstrating the inhibitory breadth of VHH5.

To understand the molecular basis underlying the broad inhibitory activity of VHH5 observed across divergent lineages, we next compared NA sequences across representative IBV strains (Fig. 6B). As described above, our protein-protein docking and MD simulations identified a binding mode in which VHH5 residues Y98, D100, Y100a, and R100b engage the conserved NA residues E373, R292, T146, and D149 within the catalytic site. Consistent with this model, NA sequence alignment revealed that these four VHH5-contacting NA residues are fully conserved across all B/Victoria/2/87-like lineage viruses (Fig. 6B). This high degree of conservation suggests that VHH5 may provide robust protection against antigenically divergent B/Victoria/2/87-like IBVs, including the newly emerged clade identified in recent surveillance.

## DISCUSSION

In this study, we isolated and characterized a series of nanobodies from an immune VHH library that inhibit IBV NA activity. Among these, VHH5 was selected as the lead nanobody based on its inhibitory activity against representative IBV NAs. Following Fc fusion, VHH5-Fc retained nanomolar inhibitory activity against the panel of IBV NAs tested in this study, including ancestral, B/Yamagata/16/88-like, B/Victoria/2/87-like, and NAI-resistant variants. In a lethal mouse challenge model, administration of either 30 μg or 100 μg of VHH5-Fc conferred full protection under a high-dose IBV challenge in both prophylactic and therapeutic settings. These findings highlight the potential of VHH5-Fc as a promising therapeutic candidate for IBV infection, offering broad and potent protection across antigenically divergent lineages.

Although VHH5-Fc protected mice from mortality in both prophylactic and therapeutic settings, some treated animals showed transient body-weight loss during the early phase after challenge (Fig. 4B and E). This finding is likely related, at least in part, to the stringency of the challenge model. In contrast to many studies of anti-influenza virus monoclonal antibodies that use a 5× mLD50 challenge dose (32, 33), we used a 25× mLD50 dose of B/Anhui/01/2024. Consistent with a previous report (34), transient early body-weight loss followed by recovery can occur under such high-dose challenge conditions. In addition, as VHH5-Fc targets NA, its protective effect is expected to act mainly by limiting progeny virus release and subsequent viral spread, rather than by completely preventing initial infection by the challenge virus. This may explain why transient weight loss was still observed despite complete or substantial protection from mortality. A strictly linear dose-response relationship was also not apparent in the survival curves within the dose range tested (Fig. 4C and F). This may reflect the limited sensitivity of survival as an endpoint once protection approaches saturation. Future studies using a broader dose range, together with additional virological and clinical readouts, will be needed to better define the *in vivo* dose-response relationship of VHH5-Fc.

Molecular dynamics simulations combined with site-directed mutagenesis revealed that VHH5 targets the catalytic site of IBV NA. Its elongated CDR3 loop penetrates deeply into the active pocket, forming stable interactions with multiple conserved residues via a “DYR” motif, thereby sterically blocking substrate access and effectively inhibiting enzymatic activity. Interestingly, the recently reported nanobody VHH525 also employs a “DYR” motif within its CDR3 to engage key residues in the catalytic region (27), suggesting a conserved mechanism of inhibition. These results underscore the functional significance of the “DYR” motif in mimicking the natural interaction between sialic acid and the NA active site. Despite this similarity, molecular dynamics analyses revealed distinct binding modes: whereas VHH525 adopts a vertical insertion conformation, VHH5 preferentially binds in a tilted orientation (pose 3) (Fig. S3). These differences may be attributed to variations in CDR loop composition, which cooperatively influence inter-loop interactions and modulate nanobody flexibility.

Recent structural studies on human broadly inhibitory antibodies against IAV NA have demonstrated that the “DR” or “RD” motifs within the HCDR3 mimic sialic acid interactions, thereby enabling cross-subtype inhibition of IAV NAs (20, 33). However, such antibodies typically show limited efficacy against IBV and often fail to achieve cross-lineage inhibition between the B/Victoria/2/87-like and B/Yamagata/16/88-like lineages. Similar to the previously reported VHH525 (27), VHH5 employs a “DYR” motif in its CDR3 to engage conserved residues in IBV NA, suggesting that this molecular recognition strategy represents a mechanism for potent and broad enzymatic inhibition. This observation suggests that the “DYR” motif may represent a sequence feature associated with targeting the catalytic site of IBV NA. More specifically, our data indicate that an elongated CDR3 loop containing acidic, aromatic, and basic residues may support interactions with conserved residues around the IBV NA active site. These findings provide a useful sequence feature for future screening or engineering of IBV NA-directed nanobodies. Nevertheless, current structural and functional evidence supporting the generality of this motif remains limited, and further identification of additional inhibitory nanobodies, together with high-resolution structural analyses of their complexes, is required to validate its broader applicability.

The recombinant NA protein used as an immunogen in this study was derived from B/Colorado/06/2017 (B/Victoria/2/87-like lineage). The elicited nanobodies exhibited cross-lineage inhibitory activity against NAs from ancestral, B/Yamagata/16/88-like, and B/Victoria/2/87-like lineages. This differs from a previous report in which an admixed multivalent immunization strategy was used to achieve cross-reactivity (27), and is consistent with previous work showing that single-lineage recombinant NA immunization can elicit cross-reactive and protective anti-NA immune responses (35). Although our immunization strategy successfully generated NA-specific nanobodies, we acknowledge that the 1 mg immunization dose used here is relatively high. Because well-established alpaca immunization protocols using recombinant IBV NA protein have not yet been defined, this dose was selected empirically to increase the likelihood of successful immunization. Future studies will be needed to optimize the immunization dose and regimen for recombinant IBV NA in alpacas. The ability of influenza B virus NA-based vaccines to protect mice and to reduce or block transmission in guinea pig models has been reported previously (35, 36). In line with these reports, our recombinant IBV NA protein elicited cross-lineage inhibitory nanobodies in alpacas, supporting the immunogenicity of this antigen under the experimental conditions used in this study. These findings suggest that this recombinant IBV NA immunogen may have potential value for NA-based vaccine-related studies and warrant further evaluation in future work.

To improve pharmacokinetic properties and enhance clinical translational potential, the VHH5 was fused to the human IgG1 Fc domain to generate the VHH5-Fc fusion protein. It is well established that Fc fusion can prolong circulating half-life and increase molecular stability (37). Although there is currently no direct experimental evidence supporting Fc-mediated steric hindrance by the VHH-Fc format blocking the NA catalytic pocket, a previous study reported that certain NA-targeting monoclonal antibodies can inhibit enzymatic activity through steric blockade mechanisms (38). Therefore, we hypothesize that the increased molecular size of VHH5-Fc may further impede substrate access to the NA catalytic site. Previous studies have also shown that fusion of nanobodies to an Fc fragment increases functional affinity through avidity effects conferred by dimeric (or multivalent) architectures (39). Consistently, VHH5-Fc exhibited improved NA inhibition activity *in vitro* compared to the monomeric form and provided effective protection in a lethal mouse challenge model. Although we did not specifically evaluate the contribution of Fc-mediated antibody-dependent cellular cytotoxicity, previous evidence has confirmed that the *in vivo* efficacy of broadly reactive anti-NA antibodies frequently depends on FcγR-mediated effector functions (18, 40). Therefore, in addition to direct inhibition of enzymatic activity, we cannot exclude the possibility that VHH5-Fc may promote viral clearance through Fc-mediated immunomodulatory mechanisms.

IBV phylogenetic analysis demonstrated continued diversification of the B/Victoria/2/87-like lineage with two monophyletic clades emerging since 2020 and increasing antigenic divergence from the current WHO vaccine strain (from 2022 to 2026, B/Austria/1359417/2021 [41]) (Fig. S4A). Such divergence raises concern that HA-mediated cross-protection may progressively diminish, posing a risk of future immune evasion. In contrast, NA remains markedly conserved. Sequence alignment showed that all four residues contacted by the VHH5 nanobody are invariant across B/Victoria/2/87-like viruses, suggesting that NA-targeting nanobodies could retain activity despite ongoing HA evolution. Furthermore, these observations also highlight NA as a conserved and promising target for broadly protective IBV interventions.

Nanobodies inherently possess excellent potential for structural engineering. Previous studies have demonstrated that multivalent or bispecific formats can enhance functional performance and improve tolerance to antigenic variation (39, 42). Leveraging this advantage, VHH5 could be engineered into bispecific or dual-epitope binding molecules by combining it with nanobodies targeting conserved epitopes of influenza virus HA or NA subtypes. Such designs aim to generate molecules capable of simultaneously binding conserved regions of both influenza A and B viruses, thereby substantially broadening the spectrum of inhibition and enhancing potency. Furthermore, existing studies have confirmed the favorable physicochemical stability of nanobodies in inhalable delivery systems (43), suggesting that VHH5 may also be suitable for development as an inhaled therapeutic agent. However, these strategies remain in the prospective stage and require systematic evaluation in future studies.

This study has several limitations. First, although the proposed VHH5-NA binding mode is supported by molecular modeling, MD simulations, and targeted mutagenesis, a high-resolution experimental structure of the VHH5-IBV NA complex, such as a cryo-EM or X-ray crystallographic structure, is not yet available. Future structural studies will be required to define the interaction at atomic resolution. Second, escape-mutant selection was not performed in this study and may provide complementary information on the functional epitope and potential viral escape pathways of VHH5. Finally, although VHH5-Fc demonstrated protective efficacy in the mouse challenge model, whether it can reduce viral transmission remains to be evaluated in an influenza B virus transmission model, such as the guinea pig model described by Pica et al. (44). These issues warrant further investigation in future studies.

In summary, we have identified a series of nanobodies targeting the catalytic site of IBV NA with broadly inhibitory activity, among which VHH5 exhibits potent inhibition. The VHH5-Fc antibody demonstrates significant antiviral efficacy in both *in vitro* and *in vivo* models. Mechanistic studies reveal that VHH5 binds conserved residues of the NA active site through a highly conserved “DYR” motif in its CDR3 region, thereby effectively inhibiting multiple antigenically distinct IBV strains. The discovery of VHH5 expands the repertoire of anti-IBV NA antibodies and supports the further development of nanobody-based antiviral therapeutics targeting IBV.

## MATERIALS AND METHODS

### Cells and viruses

HEK293T cells and Madin-Darby canine kidney (MDCK) cells were obtained from the American Type Culture Collection and cultured in Dulbecco’s modified Eagle’s medium (Gibco) supplemented with 10% fetal bovine serum (CellMax), 2 mM L-glutamine (Gibco), and penicillin (100 U/mL) and streptomycin (100 μg/mL) solution (CellMax). HEK293 cells were maintained in a serum-free, chemically defined medium (Zhuhai Kairui, Zhuhai, China). Sf9 cells were cultured in serum-free insect cell medium (SIM-SF; Sino Biological, Beijing, China) supplemented with penicillin (100 U/mL) and streptomycin (100 μg/mL) solution (CellMax). High Five cells were maintained in serum-free High Five insect cell medium (SIM-HF; Sino Biological, Beijing, China) supplemented with penicillin (100 U/mL) and streptomycin (100 μg/mL) solution (CellMax).

The influenza B virus strain B/Anhui/01/2024 was isolated from a nasal swab specimen collected from an adult patient by the Harbin Veterinary Research Institute, Chinese Academy of Agricultural Sciences. The influenza B virus strain B/Yamagata/16/1988 was obtained from the Chinese National Influenza Center, Chinese Center for Disease Control and Prevention. Both viruses were propagated in MDCK cells cultured in minimum essential medium (MEM) supplemented with bovine serum albumin (BSA) and tolylsulfonyl phenylalanyl chloromethyl ketone (TPCK)-treated trypsin (0.5 μg/mL). Following propagation using MDCK cells, virus-containing supernatants were collected, aliquoted, and stored at −80°C until further use. The B/Anhui/01/2024 virus used for mouse challenge was not mouse-adapted.

### Animals

Female BALB/c mice (strain code 000,671; Shanghai Lingchang Biotechnology, Shanghai, China) were purchased at 7 to 8 weeks of age and maintained in a specific-pathogen-free facility under controlled environmental conditions (22 ± 2°C, 40 to 60% relative humidity, and a 12 h light/dark cycle) with *ad libitum* access to sterilized food and water. A 3-year-old female alpaca used for immunization was sourced from a licensed local supplier in Harbin, China.

### Recombinant NA protein expression

Recombinant NA proteins were expressed using a baculovirus-insect cell expression system. Gene fragments encoding the head domains of representative influenza virus NA were subcloned into the pFastBac transfer vector (Invitrogen). To enhance secretion and facilitate affinity purification, an N-terminal gp67 signal peptide and an 8×His tag were fused to the NA coding sequence. Additionally, a vasodilator-stimulated phosphoprotein (VASP) tetramerization motif was incorporated to promote proper oligomeric assembly and stabilize the soluble head region in a near-native conformation. The pFastBac-gp67-8×His-VASP-NA construct was then used to generate bacmids by transforming *E. coli* DH10Bac competent cells. Recombinant baculovirus was produced by transfecting bacmids into Sf9 cells. For large-scale expression, High Five cells were infected at a multiplicity of infection of 5 and cultured at 27°C with shaking at 150 rpm. Culture supernatants were harvested when cell viability declined to approximately 60%. Recombinant NA proteins were purified by Ni^2+^-nitrilotriacetic acid affinity chromatography using His-tag purification resin (Beyotime).

### Alpaca immunization and generation of an immune VHH library

An immune VHH library was generated as previously described, with minor modifications (45). Briefly, a 3-year-old female alpaca was immunized three times at 2 week intervals with recombinant NA from B/Colorado/06/2017 (B/Victoria/2/87-like lineage) formulated with Freund’s adjuvants. The primary immunization consisted of 1 mg recombinant NA in 1 mL PBS, mixed 1:1 with complete Freund’s adjuvant (Sigma). Each booster dose contained 500 µg of recombinant NA in 1 mL of PBS mixed 1:1 with incomplete Freund’s adjuvant (Sigma). Antigen-specific serum IgG titers were determined by indirect ELISA using horseradish peroxidase (HRP)-conjugated AffiniPure goat anti-alpaca IgG (H+L) as the secondary antibody (Jackson ImmunoResearch, USA). A titer greater than 1:32,000 following the final boost indicated a robust humoral immune response.

Seven days after the last immunization, approximately 100 mL of blood was collected from the peripheral veins. PBMCs were isolated using Ficoll-Paque PLUS (TBD, Tianjin, China). Total RNA was extracted with TRIzol reagent (Invitrogen) and reverse-transcribed with oligo(dT)₂₀ primers and the PrimeScript RT kit (Takara). The VHH regions were amplified by nested PCR using alpaca-specific primer sets. In the first round, PCR amplification was performed with primers CALL001 (5′-GTCCTGGCTGCTCTTCTACAAGG-3′) and CALL002 (5′-GGTACGTGCTGTTGAACTGTTCC-3′), followed by gel extraction using a QIAquick Gel Extraction Kit. A second amplification step was then carried out using primers FR1-RSCF (5′-GAGGAGGAGGAGGAGGAGGCGGGGCCCAGGCGGCCCAGGTGCAGCTGCAGGAGTCTGGRGGAGG-3′) and VHH-RSCB (5′-GAGGAGGAGGAGGAGGAGCCTGGCCGGCCTGGCCACTAGTGGCGGCCGCTGAGGAGACGGTGACCTGGGT-3′) to obtain the full VHH fragments. The purified products were subsequently cloned into the pComb3X phagemid vector. The ligation products were electroporated into *Escherichia coli* TG1 cells (AlpVHHs, China) to construct the immune VHH library. The library was rescued by infection with helper phage M13K07 (AlpVHHs, China), and phage particles were precipitated with polyethylene glycol 8000 to generate a high-titer phage stock for subsequent panning.

### Solid-phase panning for nanobody selection

Solid-phase bio-panning was performed using high-binding 96-well ELISA plates (NEST) coated with recombinant NA from B/Colorado/06/2017 at concentrations of 8 µg per well in the first round, 4 µg per well in the second, and 1 µg per well in the third; PBS-coated wells served as negative controls. Plates were incubated overnight at 4°C, washed three times with PBS, and blocked with 5% (wt/vol) skim milk in PBS for 2 h at 37°C. Following three washes with PBST (PBS containing 0.05% Tween-20), 1 × 10¹¹ phage particles were added to each well and incubated for 2 h at 37°C. Unbound phages were removed using progressively stringent washes with PBST, followed by PBS in subsequent rounds. Bound phages were eluted with 0.1 M triethylamine (pH 12.0) for 10 min at room temperature (RT) and immediately neutralized with 1 M Tris-HCl (pH 7.4). The neutralized eluate was used to infect log-phase TG1 cells. Infected cultures were amplified and superinfected with M13K07 helper phage to facilitate the display of VHHs. Phage particles harvested from culture supernatants were precipitated and carried forward into the next round of selection.

### Screening of positive VHH clones by phage ELISA

Forty-eight colonies were randomly selected following the third round of bio-panning and cultured in 96-well deep-well plates (NEST) containing 2×YT medium supplemented with carbenicillin (100 μg/mL). Cultures were superinfected with M13K07 helper phage, selected in the presence of kanamycin (50 μg/mL), and incubated overnight at 37°C with shaking. After centrifugation, culture supernatants were collected, mixed 1:1 with 3% BSA in PBS, and used directly as primary antibodies in ELISA.

Two sets of high-binding 96-well ELISA plates (NEST) were coated overnight at 4°C with either recombinant NA (0.2 µg/well) from B/Colorado/06/2017 (B/Victoria/2/87-like lineage) or influenza B virus B/Anhui/01/2024 (8 µg/well). Wells coated with PBS alone served as negative controls. After removal of the coating solution, plates were washed three times with PBST (PBS containing 0.1% Tween-20), blocked with 5% (wt/vol) skim milk in PBS for 2 h at 37°C, and washed an additional three times. For each clone, 100 µL of phage-containing supernatant was added to antigen-coated and control wells, and the mixture was incubated for 2 h at 37°C. Bound phages were detected using an HRP-conjugated anti-M13 antibody (AlpVHHs, China). After washing, 100 μL of 3,3′,5,5′-tetramethylbenzidine (TMB) substrate was added, and the plates were incubated at 37°C for 10–15 min. The reaction was stopped by adding 50 μL of 2 M H₂SO₄. Absorbance at 450 nm was measured using a microplate reader. Data were analyzed using GraphPad Prism 10. Clones were considered positive if the OD₄₅₀ values in both rNA-FluB and virus-coated wells were at least fourfold higher than those in PBS-coated control wells. Sequence analysis of the positive clones identified six unique VHH sequences.

### Surface plasmon resonance

Binding kinetics between the IBV NA protein and VHH5 were analyzed at 37°C using a Biacore 8K surface plasmon resonance system (Cytiva). Experiments were conducted in PBST buffer (2 mM KH₂PO₄, 8 mM Na₂HPO₄, 136 mM NaCl, 2.6 mM KCl, 0.05% Tween-20, pH 7.4) with a Series S Sensor Chip CM5. The VHH5 was covalently immobilized on the chip via standard amine-coupling chemistry in 10 mM acetate buffer (pH 5.0). Serial dilutions of the recombinant NA antigen were injected over the immobilized surface, and a mock-immobilized reference channel was used for background subtraction. Following each binding cycle, the sensor surface was regenerated with 10 mM glycine (pH 2.0) to remove the bound analyte and reestablish the baseline response. Sensorgrams were globally fitted to a 1:1 Langmuir binding model to derive association (*K*_on_) and dissociation (*K*_off_) rate constants. Because NA is tetrameric, the binding stoichiometry may deviate from a simple 1:1 interaction; therefore, the equilibrium dissociation constant calculated from the 1:1 model (K_D_ = *K*_off_ / *K*_on_) is reported as an apparent binding affinity (K_D_^app^). This K_D_^app^ was used to compare VHH5-NA binding under these experimental conditions, rather than as a precise stoichiometric constant, consistent with Ramos et al. (34).

### Expression and purification of nanobodies

Six nanobodies and a bivalently derived VHH5 were expressed using a prokaryotic expression system. The coding sequences were cloned into the pET-22b expression vector (Comatebio, Changchun, China) via homologous recombination, with each construct incorporating a C-terminal 6×His tag to facilitate affinity purification. The bivalent VHH5 was constructed by linking two VHH5 domains in a head-to-tail orientation using a flexible (GGGGS)₃ peptide linker. Verified plasmids were transformed into *E. coli* Rosetta competent cells (Biomed, Beijing, China), and recombinant proteins were produced under standard induction conditions.

### Production and purification of Fc-fusion nanobodies

To generate the human IgG1 Fc-fused VHH5, the VHH5 coding sequence was cloned into an in-house mammalian expression vector. Endotoxin-free plasmid DNA was prepared and transiently transfected into suspension-adapted HEK293 cells following the manufacturer’s protocol. After 7 days of incubation, culture supernatants were collected, and the Fc-fusion proteins were purified by protein A affinity chromatography (Cytiva). Bound proteins were eluted using a descending pH gradient of glycine buffer (pH 3.5–3.0) and immediately neutralized with 1 M Tris-HCl (pH 9.0) to preserve protein stability. The neutralized fractions were concentrated and buffer-exchanged into PBS (pH 7.4) using 10 kDa molecular weight cutoff ultrafiltration units (Merck). Protein integrity and purity were assessed by SDS-PAGE, and the oligomeric state was analyzed by native PAGE.

### Influenza virus NA ELISA

High-binding 96-well half-area plates (Corning 3690) were coated with 50 µL per well of recombinant neuraminidase (NA; 2 µg/mL in PBS) and incubated overnight at 4°C. After removal of the coating solution, the plates were washed six times with PBST (PBS containing 0.1% Tween-20) and blocked with 3% BSA for 1 h at RT. Nanobody samples were initially prepared at a concentration of 10 µg/mL and serially diluted threefold in PBST supplemented with 0.05% Tween-20 and 1% BSA. A volume of 75 µL per well was added and incubated for 1.5 h at RT. Following six washes, 50 µL per well of the HRP-conjugated AffiniPure goat anti-alpaca IgG, specific for the VHH domain (Jackson ImmunoResearch, USA), diluted 1:10,000, was added and incubated for 1.5 h at RT. After six additional washes, 50 µL of TMB substrate was added to each well and allowed to develop for 5–7 min before the reaction was stopped with 50 µL of ELISA stop solution (BBI/Sangon Biotech, catalog number E661006). Absorbance was measured at 450 nm using a microplate reader. Data were analyzed using GraphPad Prism 10 to determine the minimal binding concentration or endpoint titer. The positivity threshold was defined as the mean optical density of blank wells plus three standard deviations. A VHH specific for carcinoembryonic antigen (CEA), a human tumor-associated glycoprotein unrelated to influenza virus antigens, was included as an irrelevant negative control.

### NA-MUNANA assay

Neuraminidase inhibition activity was evaluated using the NA-Fluor Influenza Neuraminidase Assay Kit (Applied Biosystems). Nanobody samples were tested in triplicate at an initial concentration of 10 µg/mL and incubated with a fixed amount of NA-VLPs in 96-well black plates (Thermo) for 1 h at 37°C. The fluorogenic substrate MUNANA was added to a final concentration of 200 µM, and the plates were incubated for an additional hour at 37°C. Reactions were terminated with the NA-Fluor stop solution, and fluorescence was measured using a Synergy H1 microplate reader (BioTek) with excitation at 365 nm and emission at 445 nm. As negative controls, a CEA-specific VHH (VHH control) was used for assays with monomeric VHH5, and a CEA-specific VHH-Fc fusion protein (Control-Fc) was used for assays with VHH5-Fc.

### Plaque reduction neutralization assay

The plaque reduction neutralization assay was conducted as previously described (1). On day 1, 5 × 10⁵ MDCK cells were seeded into six-well plates and incubated for 24 h. On day 2, 70 plaque-forming units (PFU) of B/Anhui/01/2024 virus were preincubated with either NA nanobody preparations (final concentration: 1 µM) or control antibodies in Opti-MEM supplemented with 1% penicillin-streptomycin (S/P) for 1 h at RT. Cells were washed once with PBS and then infected with 250 µL of the virus-antibody mixture per well for 1 h at 37°C, with gentle rocking every 15–20 min. Following adsorption, 2 mL of overlay medium consisting of 1×MEM, 0.67% low-melting agarose, 0.01% diethylaminoethyl, and 1 µg/mL TPCK-treated trypsin was added to each well. Plates were incubated at 37°C for 48–72 h. After incubation, cells were fixed with 4% paraformaldehyde for 20 min at RT. The overlay was then removed, and the plates were stained with crystal violet for 20 min at RT. Finally, the plates were rinsed and washed with sterile water prior to plaque enumeration.

### *In vivo* challenge

Passive transfer experiments were conducted to evaluate the prophylactic and therapeutic efficacy of VHH5-Fc in a mouse model of influenza B virus infection. All antibody preparations were diluted in 100 µL of PBS and administered intraperitoneally. Mice in the negative control group received an irrelevant human IgG. For the prophylactic study, 7- to 8-week-old female BALB/c mice were randomly assigned to four groups: VHH5-Fc at 30 µg (*n* = 12), 50 µg (*n* = 11), or 100 µg (*n* = 14), and irrelevant IgG control at 100 µg (*n* = 12). Twenty-four hours after antibody administration, mice were anesthetized with pentobarbital sodium and challenged intranasally with 25× mLD50 of influenza B virus B/Anhui/01/2024 (B/Victoria/2/87-like lineage), corresponding to approximately 5 × 10^6^ PFU per mouse. For reference, 1 mLD50 of this virus strain is equivalent to approximately 2 × 10^5^ PFU. In the therapeutic study, mice were first infected intranasally with 25× mLD50 of the same virus strain and treated 24 h post-infection with VHH5-Fc at 30 µg (*n* = 6), 50 µg (*n* = 6), or 100 µg (*n* = 7). Control animals received 100 µg of irrelevant IgG (*n* = 6). Body weight and clinical signs were monitored daily for 14 days. Mice exhibiting a weight loss exceeding 25% of their initial body weight were humanely euthanized in accordance with institutional animal welfare guidelines.

### Protein-protein docking

The initial structures of VHH5 and the IBV NA protein were predicted using AlphaFold2 (46), and the low-confidence N-terminal loop of NA was removed. To refine these models, 50 ns of implicit-solvent MD simulations were performed using the Amber22 package (47). The proteins were parameterized with the ff19SB force field, and the MD protocol followed the procedure as described previously (48). Trajectories were sampled every 50 ps, and the final frame of the production run was selected for subsequent docking. Restricted protein-protein docking was performed using the ClusPro online server to generate candidate binding poses (49).

### Molecular dynamics simulations and MM/GBSA

Explicit-solvent MD simulations were conducted to refine the protein-protein complex and evaluate its structural stability. The system was parameterized using the ff19SB force field (50), solvated in an optimal point charge water box with a 12 Å solvation buffer, and neutralized with Na^+^ and Cl^−^ ions to achieve an ionic concentration of 0.15 M. The simulation protocol followed the procedure outlined in reference 51. The binding free energy between VHH5 and NA proteins was calculated using the MM/GBSA method applied to the MD trajectory. Free energy computations were performed using the MMPBSA.py module from the Amber22 software package at a salt concentration of 0.15 M.

### Phylogenetic comparison of IBV HA and NA sequences

To compare the evolutionary relationships among IBV lineages, multiple sequence alignments of the NA and HA amino acid sequences were generated using the MUltiple Sequence Comparison by Log-Expectation (MUSCLE) algorithm. Phylogenetic trees were constructed using the neighbor-joining method in MEGA12. To assess the conservation of the NA motif that interacts with VHH5, NA amino-acid sequences from the same set of strains were aligned using the MUSCLE (v.3.8.1551) algorithm implemented in SnapGene. Default MUSCLE parameters were used unless otherwise specified. The full virus names and accession numbers are listed in Table S1.

### Quantification and statistical analysis

All data processing and statistical analyses were performed using GraphPad Prism version 10.

## Data Availability

The data generated or analyzed during this study are included in this published article and its supplemental material.

## AFTER EPUB

*[This article was published on 10 July 2026 with an error in the equal contribution footnote. The footnote was corrected in the current version, posted on 14 July 2026.]*

## SUPPLEMENTAL MATERIAL

Supplemental material jvi.00762-26-s0001.docx Fig. S1 to S4; Table S1.

ASM does not own the copyrights to Supplemental Material that may be linked to, or accessed through, an article. The authors have granted ASM a non-exclusive, world-wide license to publish the Supplemental Material files. Please contact the corresponding author directly for reuse.
