# Supplementary material for: A neuraminidase-targeted nanobody confers broad protection against influenza B virus
Source: J Virol. 2026 Jul 10;100(8):e00762-26. doi: 10.1128/jvi.00762-26 (PMC13483244; doi:10.1128/jvi.00762-26)
Supplement: Supplementary file 1 [file jvi.00762-26-s0001.docx]

**A Neuraminidase-Targeted Nanobody Confers Broad Protection Against Influenza B Virus**

**Hang Jia^1^, Chaohui Lin^2^, Yinghao Guo^1^, Weigang Cai^3^, Renfang Guan^1^, Lizhu Zhu^2^, Qian Yang^2^,** **Huihui Kong ^4*^, Bin Sun^5*^,** **Zeli Zhang^2*^, Jian Ma^1*^**

^1^ Department of Pancreatobiliary Surgery and Department of Immunology, The Second Affiliated Hospital of Harbin Medical University, Harbin Medical University, Harbin, China;

^2^Zhejiang Key Laboratory of Multi-Omics in Infection and Immunity, Center for Infectious Disease Research, School of Medicine, Westlake University, Hangzhou, China;

^3^Worldwide Influenza Centre, The Francis Crick Institute, London, UK;

^4^State Key Laboratory of Animal Disease Control and Prevention, Harbin Veterinary Research Institute, Chinese Academy of Agricultural Sciences, Harbin, China;

^5^Research Center for Pharmacoinformatics, College of Pharmacy, Harbin Medical University, Harbin, China.

Running title: **Neuraminidase-Targeted Nanobody against Influenza B**

Hang Jia, Chaohui Lin, Yinghao Guo, and Weigang Cai contributed equally to this article.

***Correspondence**

Jian Ma

Department of Pancreatobiliary Surgery and Department of Immunology, The Second Affiliated Hospital of Harbin Medical University, Harbin Medical University, Harbin, China.

E-mail: jma@hrbmu.edu.cn

Zeli Zhang

Zhejiang Key Laboratory of Multi-Omics in Infection and Immunity, Center for Infectious Disease Research, School of Medicine, Westlake University, Hangzhou, China.

E-mail: zhangzeli@westlake.edu.cn

Bin Sun

Research Center for Pharmacoinformatics, College of Pharmacy, Harbin Medical University, Harbin, China.

E-mail: binsun@hrbmu.edu.cn

Huihui Kong

State Key Laboratory of Animal Disease Control and Prevention, Harbin Veterinary Research Institute, Chinese Academy of Agricultural Sciences, Harbin, China.

E-mail: konghuihui@caas.cn

1. **Supplementary figures**

**Supplementary Figure 1. Screening of nanobodies targeting influenza B virus neuraminidase.**

**(A)** SDS-PAGE followed by Coomassie Brilliant Blue staining showing the expression and purification of with recombinant neuraminidase (rNA-FluB) derived from B/Colorado/06/2017 (B/Victoria/2/87‑like lineage). **(B)** Serum antibody titer against rNA-FluB after immunization procedure. **(C)** Enrichment of NA-specific phage clones during bio-panning. **(D)** Expression and purification of anti-NA VHHs analyzed by SDS-PAGE. **(E)** The apparent binding affinity of VHH5 to rNA-FluB was measured by surface plasmon resonance (SPR) **(F)** NA inhibition curves of VHH5-Fc against NA-VLPs derived from Wuhan spiny eel influenza virus (WSEIV) NA were measured using the MUNANA assay.

**Supplementary Figure 2. Computational evaluation demonstrates that the Pose-3 binding model is applicable across different influenza B virus lineages.**

(A) Residue-wise contribution of VHH5 in the Pose3 conformation calculated by MM/GBSA. (B) RMSD trajectories of the VHH5 complexes with NA proteins from different lineages in Pose3 binding mode. (C) The Binding free energy of the VHH5-NA complexes was calculated by MM/GBSA.

**Supplementary Figure 3. Comparative analysis of the binding modes and energetics of VHH5 and VHH525 with influenza B neuraminidase.**

(A) Comparison of the binding modes of VHH5 (red) and VHH525 (white) with the NA protein. (B) Buried solvent-accessible surface area (SASA) of VHH5, VHH525, and VHH5 aligned in the VHH525 binding mode were calculated using Cpptraj during MD simulations. (C) Binding free energies of VHH5, VHH525, and VHH5 aligned in the VHH525 binding mode were computed by the MM/GBSA method.

**Supplementary Figure 4. Phylogenetic diversification of IBV lineages.**

(A) Divergent evolutionary trajectories of HA sequences from representative influenza B viruses, including WHO-recommended vaccine strains, historical evolutionary intermediates, and recently emerged variants. The phylogeny resolves three major groups—B/Yamagata/16/88-like (blue), the B/Victoria/2/87‑like (red), and early diverged/ancestral strains (black). Scale bar represents amino-acid substitutions per site.

2. Supplementary tables

Supplementary table 1. Listing of influenza B virus strains and NA/HA sequence accession numbers used in this study.

| **Virus strain** | **Accession Number** | **Gene segment** |
| --- | --- | --- |
| B/Ghana/3778/2024 | EPI_ISL_19513128 | NA and HA |
| B/Colorado/06/2017 | EPI_ISL_257735 | NA and HA |
| B/Washington/02/2019 | EPI_ISL_341131 | NA and HA |
| B/Switzerland/329/2024 | EPI_ISL_18950190 | NA and HA |
| B/Phuket/3073/2013 | EPI_ISL_166957 | NA and HA |
| B/Austria/1359417/2021 | EPI_ISL_1519459 | NA and HA |
| B/Stockholm/3/2022 | EPI_ISL_13817451 | NA and HA |
| B/Michigan/01/2021 | EPI_ISL_965138 | NA and HA |
| B/Brisbane/60/2008 | EPI_ISL_246494 | NA and HA |
| B/Greece/5509/2024 | EPI_ISL_18950198 | NA and HA |
| B/Yamagata/16/1988 | NCBI_AAN39803.1 | NA |
| B/Yamagata/16/1988 | EPI_ISL_13660164 | HA |
| B/NewYorkCity/PV00094/2017 | NCBI_MW651797.1 | NA |
| B/NewYorkCity/PV00094/2017 | NCBI_MW651799.1 | HA |
| B/Lee/1940 | EPI_ISL_115140 | NA and HA |
| B/Victoria/2/1987 | EPI_ISL_13660159 | NA and HA |
| B/Norway/07673/2024 | EPI_ISL_19723813 | NA and HA |
| B/Yekaterinburg/37/2024 | EPI_ISL_19558694 | NA and HA |
| B/Novorossiysk/RII-01/2024 | EPI_ISL_19558692 | NA and HA |
| B/SaintPetersburg/RII-51/2024 | EPI_ISL_19558693 | NA and HA |
| B/Kazan/RII-01/2024 | EPI_ISL_19558685 | NA and HA |
| B/Catalonia/NSVH102439757/2024 | EPI_ISL_19558676 | NA and HA |
| B/HongKong/8/1973 | P16191 | NA |
| B/HongKong/8/1973 | P03462 | HA |
| B/Anhui/01/2024 | EPI_ISL_20254957 | NA and HA |
